# Editorial: Abstract Mathematical Cognition

**DOI:** 10.3389/fnhum.2015.00719

**Published:** 2016-01-26

**Authors:** Philippe Chassy, Wolfgang Grodd

**Affiliations:** ^1^Mathematical Cognition Research Group, Department of Psychology, Liverpool Hope UniversityLiverpool, UK; ^2^Department of High Field Magnetic Resonance, Max Planck Institute for Biological CyberneticsTübingen, Germany

**Keywords:** mathematical cognition, abstract concepts, learning, developmental psychology, expertise development

Despite the importance of mathematics in our educational systems, little is known about how abstract mathematical thinking emerges. Most research on mathematical cognition has been dedicated to understanding its more simple forms such as seriation and counting. Although these forms constitute the foundational plinth upon which all other maths skills develop, the gap between basic skills and the processing of complex mathematical concepts is poorly understood. What has come to be sufficiently well understood, however, is how numeracy is acquired. The 90s marked a change in our approach to human cognition in general and to mathematical cognition in particular. Neuroimaging technologies have enabled localization of neural activity, revealing that mathematical cognition, like other forms of cognition and skills, depends upon a network of activation. The key finding from neuroimaging and single cell recording is that numerical information is held in the intraparietal sulcus. Now that the core of mathematical cognition has been identified it is time to understand how basic skills are used to support the acquisition and use of abstract mathematical concepts. Chassy and Grodd (2012) opened the door for abstract mathematical cognition by examining for the first time the neural correlates of negative numbers, an abstract mathematical concept that emerges early on in mathematical curricula. The present issue reports crucial advances in our understanding of the neural underpinnings of abstract mathematical cognition.

For a general introduction to the topic the reader is referred to the article signed by Moeller et al. The article offers an excellent overview of the networks that are involved to some degree in processing quantities, the very basis of mathematical cognition. The authors' conclusion strengthens the view that a frontal parietal network constitutes the essence of our abilities in mathematics. The fronto-parietal network has been highlighted by a number of studies and is thought to underpin the learning of mathematical concepts. By increasing the complexity of the concepts stored in our memory, we improve the quality of our understanding of the physical world in the first stages of mathematical cognition. Abstract concepts are then able to emerge from concrete, physical quantities.

On the path of mathematical development, the first step toward an abstract representation of concepts is the shift from concrete, object-based cognition to the use of symbols. The symbols, though arbitrary, represent concrete quantities that help children quantify and thus understand the world around them. Roesch and Moeller support this view by suggesting that an internal representation of fingers contributes to the actual ability to represent quantities. In a similar vein, a cross cultural study authored by Bender and Beller compares the Western counting system to a Polynesian language of the Tonga island, offering a unique view of how concrete counting of different objects leads to an abstract representation of numbers; thus demonstrating that the roots of abstract mathematical cognition emerge from basic, sensory abilities (a long standing view that finds a new echo here). By highlighting the concrete roots of mathematical cognition, the authors of these studies open the debate on the inheritance of mathematical skill by pointing toward very concrete sensory performance.

The symbols in a later stage of mathematical development are used to represent concepts of an abstract nature. That is, once the notion of natural number is acquired, the next step toward expertise is to formalize operations as abstract entities. For example, the operation 5 + 4 = 9 is concrete and can be taught by using objects. Dowker demonstrates that pupils tend to use the same problem-solving strategies to solve problems in subtraction and addition problems. Since the properties of the two operations differ the application of the same strategy leads the pupil to commit errors. Pupils have to learn a new set of properties to be able to solve subtraction. Similarly, Huber et al. argue that mental representations of fractions do not differ from natural numbers; what do differ are the strategies used to encode information. Dowker's and Hubet et al.'s views are in line with the study of Mihulowicz et al. who, by comparing left and right lesioned patients, showed that arithmetic operations are underpinned by different networks. The view of some educators, that subtraction and addition are mirror operations, is mistaken. It is interesting to note that teaching might be adapted so that different approaches could be used to teach different operations. The studies highlight the fact that learning arithmetic includes knowledge that is not purely numerical. This is our first hint indicating that educational strategies might have a huge influence on the ability of students to learn abstract concepts. The next stage in mathematical learning is the step consisting in moving from concrete (arithmetic) to abstract (algebraic) relationships. A study by Susac et al. looked at this move and showed that it requires about 4 years of training to master this new step toward abstract thinking in mathematics. It is crucial to note that these 4 years are in addition to the many years required for correctly mastering the basics. Mathematical learning is a long road. It calls for pedagogical approaches that are specific to each level.

Two main variables might modulate the acquisition of mathematical expertise: Educational system and inherited factors. The idea that teaching practices impact heavily on the ability of students to develop their skills in abstract mathematical cognition is demonstrated by Prado et al. The authors ran a cross cultural study comparing Chinese and American students on problem-size effects, and show that educational practices, which differ in the 2 countries, impact on the wiring of the network in charge of symbolic arithmetic. In line with this result, McLean and Rusconi attempt to bridge the gap between the findings of academic science and the practical problems faced by teaching institutions when dealing with students with mathematical difficulties. After revealing the cognitive factors underpinning the acquisition of mathematical knowledge, McLean and Rusconi discuss the types of interventions that may help students with mathematical difficulties. With respect to inherited factors, Zhang et al. have shown that gifted adolescents display a highly integrated fronto-parietal network, hence displaying a more efficient link between the representation of numbers in the parietal cortex and working memory in the prefrontal cortex.

The many findings of the articles in this special topic call for further research to see how specific neural networks serve various abstract mathematical concepts.

## Conflict of interest statement

The authors declare that the research was conducted in the absence of any commercial or financial relationships that could be construed as a potential conflict of interest.

## References

[B1] Chassy P. Grodd W. (2012). Comparison of quantities: core and format-dependent regions as revealed by fMRI. Cereb. Cortex 22, 1420–1430. 10.1093/cercor/bhr219 21878489

